# Breaking the vicious cycle of delayed healthcare seeking for people who use drugs

**DOI:** 10.1186/s12954-025-01166-3

**Published:** 2025-03-05

**Authors:** Zoi Papalamprakopoulou, Elisavet Ntagianta, Vasiliki Triantafyllou, George Kalamitsis, Arpan Dharia, Suzanne S. Dickerson, Angelos Hatzakis, Andrew H. Talal

**Affiliations:** 1 Division of Gastroenterology, Hepatology, and Nutrition, UB-CTRC, Jacobs School of Medicine and Biomedical Sciences, University at Buffalo, State University of New York, Suite 6090, 875 Ellicott Street, Buffalo, NY 14203 USA; 2 Hellenic Scientific Society for the Study of AIDS, Sexually Transmitted and Emerging Diseases, Athens, Greece; 3 Hellenic Liver Patient Association “Prometheus”, Athens, Greece; 4 Faculty Development and PhD Program, School of Nursing, University at Buffalo, Buffalo, NY USA; 5 Department of Hygiene, Epidemiology and Medical Statistics, National and Kapodistrian University of Athens, Athens, Greece

**Keywords:** Healthcare access, Healthcare barriers, People who use drugs

## Abstract

**Background:**

People who use drugs (PWUD) are at increased mortality risk, yet they typically avoid healthcare settings due to stigma and shunning. Understanding the healthcare journey from the viewpoint of PWUD has been understudied, although it is essential for informing solutions to increase healthcare access to improve their healthcare outcomes. We aimed to understand the process of accessing healthcare for PWUD, including perceived barriers and facilitators, by exploring their experiences, attitudes, and beliefs.

**Methods:**

We employed purposive sampling to recruit PWUD to participate in nine focus group discussions (FGDs) (N = 57) in Athens, Greece. Inclusion criteria required a history of injection drug use, internet access, and Greek verbal fluency. The FGDs were audio-recorded, transcribed, translated into English, and de-identified. We analyzed FGD transcripts using modified grounded theory.

**Results:**

Participants’ mean (standard deviation) age was 47.9 (8.9) years, 89.5% (51/57) were male, 91.2% (52/57) were of Greek origin, and 61.4% (35/57) had attended at least 10 years of school. We identified three key themes from the FGD transcript analysis: (1) seeking care after an individual’s rapid health decline, (2) facing barriers in accessing healthcare, and (3) building trust to improve access to healthcare for PWUD. Participants disclosed that they tended to seek healthcare after a rapid deterioration in their health. They experienced multiple barriers to healthcare access such as stigma, healthcare system mistrust, unresponsive emergency medical services and competing priorities such as homelessness, mental health challenges, and ongoing manifestations of substance use disorder (SUD). Participants’ recommendations to build patient-provider trust and improve healthcare access include stigma minimization, promotion of empathy in the patient-provider relationship, and engaging community organizations that serve PWUD to build bridges with healthcare providers and institutions.

**Conclusions:**

PWUD in Athens, Greece demonstrate delayed health-seeking behaviors and report multifaceted healthcare access barriers including stigma, delays in emergency care, poor mental health, homelessness, and SUD manifestations. Key trust-building processes to expand healthcare access include minimizing stigma and promoting empathy in healthcare encounters, enhancing healthcare staff education on SUD, improving the responsiveness of emergency medical services, engaging community organizations, and exploring telehealth’s role in improving healthcare access for PWUD.

**Supplementary Information:**

The online version contains supplementary material available at 10.1186/s12954-025-01166-3.

## Introduction

People who use drugs (PWUD) face increased mortality rates worldwide when compared to the general population, with major causes of death including overdoses, suicide, violence, accidents, HIV, and comorbid health conditions such as cardiovascular disease [1]. These conditions may be exacerbated in PWUD since the population often faces limited healthcare access, creating significant barriers to receiving necessary care and accessing highly effective treatments for conditions such as HIV and hepatitis C virus (HCV) infections [2–5]. The restricted healthcare access for PWUD highlights the inadequacies of the healthcare delivery system to address their complex needs, leading to poor health outcomes [5]. Stigma within the healthcare setting is the most well-described barrier to accessing care for PWUD [2]. Beyond stigma, PWUD have described additional healthcare access challenges, including limited health literacy, de-prioritization of medical care, and difficulty navigating the healthcare system [3, 4].

While a body of evidence has identified some of the barriers to healthcare access, gaining further insight directly from the perspectives of PWUD is crucial to identify approaches to expand healthcare access. Understanding PWUDs’ perceptions of factors inhibiting healthcare access is a prerequisite for guiding healthcare system reforms aimed at improving health outcomes for PWUD. Additionally, in settings where healthcare is primarily government-supported, such as in Europe, a paucity of data exists on PWUD interactions with government-administered healthcare systems. Understanding these interactions is essential for informing policymakers about optimal approaches to enhance the inclusion of PWUD into healthcare in such settings. Given a paucity of research on PWUD attitudes toward accessing healthcare, we conducted our investigation in Greece. Furthermore, recent studies have reported an increasing mortality trend among Greek PWUD, emphasizing the need for targeted interventions to improve healthcare access [6].

In Greece, healthcare is delivered through a hybrid system involving both public and private providers [7]. The National Healthcare System is funded by taxes and social insurance, and it offers free or affordable access to healthcare services with several advantages [8, 9]. For example, access to specialist care can be sought even without a referral from a primary care physician, in contrast to many other healthcare systems where such referrals are required. Access to emergency medical support is facilitated by calling the emergency contact number 166 or the European emergency phone number 112, which dispatches the nearest public ambulance unit. Emergency care is also provided free-of-charge at public hospitals [10]. PWUD primarily access healthcare through the National Healthcare System. Healthcare services are also accessible free-of-charge through specifically designated shelters for PWUD experiencing homelessness, along with substance use disorder (SUD) treatment and social support [11].

We aimed to explore the process of how PWUD in Greece access healthcare, including a focus on the barriers and facilitators to the process. Engaging PWUD as research participants offers firsthand, comprehensive insights into their experiences, perspectives, and beliefs about healthcare access, as well as the social processes underlying these attitudes. This approach can guide the development of targeted interventions to expand healthcare access specifically tailored to address PWUDs’ needs.

## Methods

### Study population and recruitment

We employed purposive sampling to recruit participants [12, 13]. We contacted influential colleagues from community organizations that serve PWUD, including the Hellenic Liver Patient Association, “Prometheus”, “My Athens” (a shelter for PWUD experiencing homelessness), “Positive Voice” (an association of people living with HIV), and the “Network of Peer Users of Psychoactive Substances” to serve as facilitators for participant recruitment. The facilitators were employed by the aforementioned community organizations and possessed bachelor’s degrees in psychology or social work. The facilitators were educated on the details of the study by the research staff. We identified prospective participants through their responses to in-person announcements or through social media posts disseminated by the community organizations. The research study manager (Z.P.) contacted potential participants, either directly or through a warm hand-off process mediated by a facilitator. Inclusion criteria for participation included age ≥ 18 years, history of injection drug use, current internet access, Greek verbal fluency, and the ability to provide informed consent.

### Data collection

We selected focus group discussions (FGDs) as the most appropriate data gathering method, given their efficacy to facilitate an understanding of social issues [14]. The dynamic group process of FGDs, compared to one-on-one interviews, contributes to idea generation by promoting peer dialogues, brainstorming, and encouraging all participants to share their approaches and attitudes towards accessing healthcare [15, 16]. We did not anticipate significant distress or harm resulting from participation in the FGDs and referrals to support services were not included as part of the study [17]. However, we provided participants with contact information for relevant support services upon request for those individuals who expressed a desire.

Between May and September 2023, we conducted 9 FGDs, each comprised of 4 to 9 participants (N = 57). There were no participants who prematurely discontinued the FGDs. The FGDs were conducted by the research study manager (Z.P.) along with the co-principal investigator (A.H.), who had received training in qualitative interviewing and FGD coordination from an expert in qualitative research (S.S.D.). Each FGD lasted up to 90 min and took place in a private room in the premises of “Prometheus”. Additionally, we encouraged participants to use pseudonyms during the discussions to further protect their identities.

We developed a semi-structured interview guide containing open-ended, non-leading questions to facilitate discussions about healthcare access. Example questions included: “Please describe how do you find a doctor when you have a medical problem?”, “Please tell me about the process of accessing healthcare?”, and “Please describe the challenges and barriers that interfered with your ability to obtain medical care”. An additional file contains the complete interview guide [see Additional file 1]. If participants did not mention these aspects during the open-ended inquiry, we subsequently employed probes to further elaborate on their approaches to accessing healthcare. Prior to deployment of the interview guide, we asked the study facilitators for their critical appraisal of the cultural and literary relevance of the interview guide for our study population. All facilitators agreed on the guide’s comprehensibility and appropriateness. We also pilot tested the interview guide in one pilot FGD with four study-eligible participants, following the planned study process for recruitment, enrollment, and informed consent. After the pilot FGD, we made minor literary modifications to the interview guide to ensure further clarity and comprehensibility by the study population.

The FGDs were audio-recorded, transcribed verbatim, and translated into English by a professional agency. Field notes were also made during the FGD. The FGD transcripts were verified for accuracy by the research project manager and interviewer (Z.P.) through comparison with the recordings. Subsequently, the FGD transcripts and field notes were de-identified and shared with the research team. During the de-identification process, participants’ real names or any pseudonyms mentioned in the FGDs were replaced with labels indicating the focus group they participated in and their seating arrangement during the discussion. For example, a participant labelled “09–02” took part in the ninth focus group and was assigned the number 02 based on their seating order. At the conclusion of each FGD, we collected participants’ demographic information, including self-reported age, sex, ethnicity, and educational level. We provided participants with lunch and 10-euro compensation for their participation.

### Analysis

We employed modified grounded theory as the most suitable approach to analyze the FGDs and identify the specific processes that PWUD use to access healthcare. Employing a modified grounded theory approach, we integrated existing theoretical knowledge about the challenges experienced by PWUD in accessing healthcare, as identified in prior research, while maintaining flexibility to allow new themes to emerge through data analysis [18–21]. In parallel with conducting FGDs, we used constant comparative analyses to inform subsequent data collection. As new findings emerged on the topic of healthcare access among PWUD, we recruited additional participants for the FGDs to delve into the emerging concepts.

The iterative analysis process followed a deductive approach, involving initial independent coding by each analyst, which included preliminary themes and initial quotes. The initial analysis team consisted of the research study manager (Z.P.), co-investigator (A.D.), and the principal investigator (A.H.T.), who conducted the initial thematic coding and analysis of the FGD transcripts and accompanying field notes. Subsequently, an expert in qualitative methodology (S.S.D.), along with the co-principal investigator (A.H) joined the final analysis team to offer their opinions on the appropriateness of the analysis. The co-principal investigator possesses extensive cultural expertise from significant research experience working with PWUD. These findings were subsequently shared among the members of the qualitative data analysis team during weekly meetings. During these meetings, the analysts reviewed the FGD transcripts and field notes, discussed their findings, and compared them with previous transcripts. Any disagreements between the analysts were discussed until consensus was reached; if no consensus was reached, the original transcript text was revisited. The full five-member analysis team conducted the final coalescence of themes. When no new insights emerged from the analysis of the FGD transcripts, we determined that we had achieved saturation and concluded further data collection. The study adheres to the consolidated criteria for reporting qualitative research (COREQ) [see Additional file 2] [22].

### Ethics

The study protocol was approved by the Hellenic Scientific Society for the Study of AIDS, Sexually Transmitted and Emerging Diseases and the University at Buffalo Institutional Review Boards. The study adheres to the principles outlined in the Helsinki Declaration. We obtained written informed consent from all eligible participants prior to their participation and encouraged confidentiality among participants. Additionally, we reconsented participants verbally before the recording started, which was documented in the FGDs’ audio recording.

## Results

### Participant characteristics

**Fig. 1:**
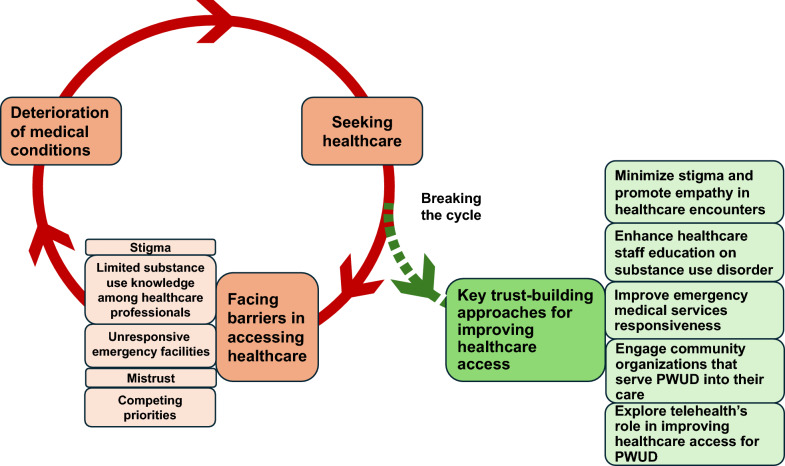
Breaking the vicious cycle of delayed healthcare seeking for people who use drugs. PWUD, people who use drugs

Participants had a mean age with a standard deviation of 47.9 (8.9) years and 89.5% (51/57) were male. The majority (91.2%, 52/57) were of Greek origin and 61.4% (35/57) had attended at least 10 years of school. We identified three key themes from the FGD transcript analysis: (1) seeking care after an individual’s rapid health decline, (2) facing barriers in accessing healthcare, and (3) building trust to improve access to healthcare for PWUD. Figure 1 illustrates the key themes that emerged from the FGDs (Fig. 1).

Our findings indicate that PWUD primarily seek healthcare when their medical conditions deteriorate. However, PWUD face various barriers when attempting to access healthcare, which further worsens their health outcomes. This situation creates a vicious cycle where PWUD’s delayed healthcare-seeking behavior is exacerbated by the barriers to healthcare access, and these same barriers further challenge their healthcare-seeking journey. Simultaneously, PWUD highlight the importance of building trust to improve their healthcare access, opening a potential avenue to break the vicious cycle of seeking healthcare. As strategies to increase trust in healthcare providers and institutions, in addition to minimizing stigma, our participants emphasized the need to enhance healthcare professionals’ education on SUD and to substantially decrease the response time of emergency medical services, which often cause significant delays in patient care. Participants also expressed a preference for receiving care in community settings compared to hospitals. Lastly, the widespread use by participants of the internet for scheduling appointments and identifying providers suggests an opportunity to explore telehealth solutions as means to improve healthcare access for this population.

### Theme 1: seeking care after an individual’s rapid health decline

When we initially asked participants about their healthcare experiences, most shared stories of seeking medical attention after a rapid deterioration in their health, with the potential to lead to life-threatening events. Participants described a range of medical conditions, such as esophageal bleeding, gastric perforation, overdoses, amputations, recurrent pleural effusions, myocardial infarctions, and strokes. We observed that the life-threatening nature and severity of these health conditions was participants’ main motivator to seek healthcare. One participant shared: *“I had a problem with my stomach, which had burst, and I went to the hospital … gastric perforation”* [participant 09–01].

In their journey to address mostly critical healthcare conditions, participants employed diverse approaches for accessing healthcare. Some preferred traditional methods, such as calling emergency contact numbers and visiting the emergency department, following standard protocols *“like all citizens”*. Other participants, who were receiving treatment for SUD in opioid agonist treatment (OAT) programs, were able to receive medical referrals through the program. Participants experiencing homelessness described monthly visits to government-appointed doctors at community clinics, which served as medical units offering primary care, free-of-charge for shelters’ beneficiaries. Additionally, nursing care was available at the shelters, as mentioned by one participant: *“There are nurses who work there [at shelters for PWUD experiencing homelessness] and take care of mild pathological conditions”* [participant 06–01].

Moreover, several participants described the process of obtaining healthcare access through the internet. For example, several participants highlighted using the internet for booking physician appointments. They valued the convenience and simplicity of the internet as a practical tool to increase and facilitate healthcare access. When asked about venues where they access healthcare, one participant mentioned using the internet to schedule appointments with private providers, stating: *“Depending on the problem, I will go online and find the right doctor, I will make an appointment, and he will examine me”* [participant 04–07]. When we asked participants for more precise details on how specifically they identified the ‘right doctor’ through the internet, they mentioned using *“key words”* in internet search tools. For instance, one participant described the process of finding the right healthcare provider as follows: *“I usually Google the medical specialty I am interested in, or if I don’t know the specialty, I type some key words and I find it”* [participant 04–01]. Overall, participants described the internet as a component of their approach to identify healthcare providers and they generally expressed comfort with this method.

### Theme 2: facing barriers in accessing healthcare

Participants revealed encountering numerous barriers when accessing healthcare. Most participants described substantial delays in accessing healthcare that were revealed through unfavorably long wait times, especially when they sought medical assistance in public hospitals. A participant reported significant delays in scheduling medical appointments at public hospitals, stating: *“For a simple cough, you might wait for months”* [participant 02–07]. Another participant described their long emergency department wait times as: *“I would have to wait for at least eight or ten hours”* [participant 06–04]. Moreover, many participants disclosed instances where the emergency contact numbers would not respond to their calls. For instance, one participant shared: *“I was in a horrible condition, and I was calling the hospitals, the emergency assistance would not come”* [participant 02–04].

Even upon gaining access to healthcare, several participants reported challenges related to the quality of healthcare received. Participants perceived as indicators of poor quality healthcare venipuncture difficulties, inadequate sterilization techniques that ultimately led to amputations, and the prescription of incorrect medications for their healthcare conditions. Participants partially attributed these misdeeds to a larger issue of the lack of knowledge of SUD among healthcare providers. These experiences stoked frustration and anger amongst participants. One participant shared: *“I went to have an implant inserted, something was not sterilized as it should, by a doctor’s mistake, and I got a germ … they wanted to cut my leg”* [participant 05–07]. Another participant shared: *“They [healthcare providers] would give me medicines … which had a contraindication for patients with hepatitis, and I was a patient with hepatitis”* [participant 01–02]. Another participant stated: *“What I don’t like, and I encounter all the time is that they [healthcare providers] are not knowledgeable about drugs, nor have they tried to learn”* [participant 01–01].

The experience of stigma in the healthcare setting emerged as a commonly reported obstacle to healthcare access. Participants perceived stigma in various forms, including healthcare providers’ refusal to provide treatment based on their history of substance use, inattention, mistreatment, and mistrust by healthcare providers. One participant expressed: *“They [healthcare providers] didn’t care that much … they would say ‘he’s a junkie’”* [participant 05–05]. Another participant shared: *“They [healthcare providers] talk badly to you … don’t pay attention to you”* [participant 04–03]. Experiencing stigma led to feelings of devaluation, as mentioned by a participant who felt dehumanized and mistreated: *“Most of the time, they [healthcare providers] look at you like you are not human, but an animal”* [participant 02–03]. Participants also disclosed feeling guilty or ashamed about their lives’ journeys, which they attributed to their SUD. For instance, one participant shared: *“All those years, I have felt guilty for not contributing anything in my life. I have not worked to contribute to anything.”* [participant 01–01].

Other participants explained how they internalized the stigma encountered, and the manner in which they felt responsible for their own health struggles. They perceived that the difficulties they encountered in healthcare settings were directly related to their inappropriate or rude behavior, and, as such, were well deserved. For instance, one participant stated:

*We [PWUD] also need to make an effort… I mean, it is one thing to go to a shop to buy a shirt and say ‘hi’ to the saleswoman … just ‘Give me that’ and it’s another to say, ‘Good morning, do you have this size?’* [participant 03-02]*.*

To cope with stigma, some participants chose nondisclosure of SUD in healthcare settings, invoking alternative approaches, such as the use of nicknames instead of providing their real names. For instance, one participant described discrimination as: *“I never say I am an addict … Because I know very well, I have seen it happening with dozens of my friends, so I don’t tell them anything”* [participant 03–01].

Moreover, participants shared that addressing other life factors, including homelessness, poor mental health, SUD and withdrawal, complicated the healthcare seeking process. A participant experiencing homelessness emphasized: *“A homeless person wakes up in the morning and has to deal with other priorities”* [participant 09–03]. Participants agreed that addressing issues related to SUD and withdrawal acted as competing priorities to addressing other healthcare issues. For instance, a participant said: *“You have other priorities. You are not going to bother about your health. The only thing that matters is how to use drugs”* [participant 06–04]. Another participant stated: *“Withdrawal can make you do a lot of things. And the people [healthcare providers] need to be patient. They don’t know that you need your fix, and everything gets on your nerves”* [participant 09–08]. Participants experiencing poor mental health expressed feeling overwhelmed and found it difficult to prioritize their healthcare needs. For instance, a participant with hepatitis and mental health issues shared how they became suicidal: “*When I got out (of jail), I started shooting heroin … I got hepatitis … I have overdosed many times … because I was tired, I wanted to go away [die] like that”* [participant 08–02].

Additionally, we captured how participants’ experiences and attitudes of mistrust with confidentiality, privacy, and security within healthcare influenced their trust. Most participants expressed ambivalence and hesitation to completely trust healthcare providers, such as the following participant’s perspective on confidentiality, privacy, and security in healthcare: “*I don’t feel trust …There are things I keep to myself, and I cannot even say to God? He is just a doctor, a man, after all. I don’t know him; he doesn’t know me”* [participant 05–07].

Lastly, many participants mistrusted governmental provisions for healthcare, perceiving that the Greek healthcare system is inferior to that of other countries. Participants felt that Greece has excellent doctors, although they are constrained by limited resources, as shared by the following participant: *“We have great doctors in Greece, but the government doesn’t provide them what they need”* [participant 09–06].

### Theme 3: building trust to improve access to healthcare for PWUD

Throughout the FGDs, participants highlighted various trust-building processes in healthcare. As a consensus, participants expressed the need to minimize stigma in doctor-patient relationships. For instance, a participant emphasized the need to treat PWUD as any human being and that SUD should be treated as any other medical condition with a biological basis:

*I want to stress that we should treat drug users like human beings, and they [healthcare providers] should handle them the way they handle an old man who has a heart attack at his house, they should go and pick them up immediately* [participant 07-02].

Another participant requested doctors to *“not to put labels on others. To show respect. To apply what they have learned from the books”* [participant 02–07]. Similarly, another participant expressed: *“They [healthcare providers] should respect us for any problem we have, like I respect them as doctors” [participant 02–05].* Participants also acknowledged the positive impact of the Greek government and the internet on HIV stigma reduction. One participant explained the long process of minimizing HIV-associated stigma in Greece as follows:

*The internet and the government have helped with HIV programs … so people now know how it is transmitted and what is going on, their attitude is different now, you say ‘you are HIV positive’, and they are like, ‘okay, it’s like you have diabetes’* [participant 03–05].

Empathy in the patient-provider relationship emerged as key to building trust within healthcare settings. Participants perceived empathy as an understanding of their needs by their healthcare providers, based upon provider-patient interactions. For example, a participant perceived empathy as the healthcare provider’s interest in their problem: *“I feel his [healthcare provider’s] interest … I can see that some doctors really care and want to deal with my problem, while others, not so much”* [participant 06–03]. While most participants highly valued empathy, some placed greater emphasis on the perceived quality of medical care. For example, one participant explained: “*The way the doctor behaves is important, but on the other hand, I think that I am more interested in the way he treats me medically than in the way he talks to me*” [participant 06–04].

Participants disclosed feeling safe in government-supported community settings, such as OAT programs and shelters. They expressed a feeling of belonging in those environments where they felt that others understood their issues and SUD treatment approaches. For instance, one participant expressed: *“To me, OKANA [OAT program] is a refuge”* [participant 03–01]. Furthermore, participants shared that they had established trusting relationships with the staff at community organizations. When medical care was facilitated through these settings, the trust engendered by the community organizations’ caregivers extended to healthcare providers and institutions. One participant mentioned: *“I have this social worker who is at OKANA … and I have a very good relationship. When I need something, she refers me to doctors”* [participant 05–04]. Another participant shared: “*Now that I have joined certain organizations, they are working with specific doctors and hospitals, I feel much better than in the past, being part of a program”* [participant 04–01].

## Discussion

Our study aimed to explore the process of healthcare access for PWUD. We identified the following three key themes from the analysis of the FGD transcripts: (1) seeking care after an individual’s rapid health decline, (2) facing barriers in accessing healthcare, and (3) building trust to improve access to healthcare for PWUD. In our study sample, PWUD sought healthcare primarily after medical conditions reached critical, life-threatening levels, a finding consistent with prior studies [23]. When attempting to access healthcare, they described facing numerous barriers, further worsening their health outcomes. This situation creates a vicious cycle where PWUD’s delayed healthcare-seeking behavior is exacerbated by the barriers to healthcare access, and these same barriers further challenge their healthcare-seeking journey.

Experiences of stigma within healthcare, including self-stigma (*i.e*. feelings of guilt and internalized shame due to a history of substance use), were among the most pervasive barriers that influenced our participants’ healthcare decisions, consistent with other studies [2, 24]. As such, PWUD often mistrust healthcare providers and institutions. As a stigma-avoidance mechanism, some participants chose not to disclose their SUD history in healthcare settings, a common approach among PWUD. For instance, a study involving 6 FGDs that included 23 PWUD enrolled in OAT programs indicated that they frequently conceal their SUD history in healthcare settings [25]. In our study, participants described experiencing inadequate responsiveness from emergency medical services, which they attributed to stigma related to SUD. They frequently reported that ambulance services would often fail to arrive promptly, resulting in significant delays in receiving necessary care. Thus, one of the important aspects to improve healthcare for PWUD is to improve emergency responsiveness especially when people are in life-threatening conditions.

Another frequently reported barrier was the perceived poor quality of healthcare that participants attributed to the inadequate knowledge of SUD by healthcare providers and staff. Manifestations of limited physician experience with SUD included unawareness of withdrawal symptoms and an inadequate understanding of the social and behavioral factors associated with SUD, such as homelessness and poor mental health. Our study participants emphasized prioritizing the avoidance of withdrawal symptoms over addressing medical issues. These results are consistent with findings from a recent study indicating inadequate withdrawal management as a major contributor to PWUD leaving or being discharged from the hospital against medical advice [26]. Moreover, the additional challenges posed by homelessness and poor mental health, as described by our participants, further emphasize the importance for healthcare providers to understand how these factors impact healthcare for PWUD. Beaulieu et al. [27] also underscored this need in their study among 1529 PWUD, demonstrating that homelessness and major depressive disorder were significantly associated with healthcare access difficulties.

While our participants expressed trust in government-supported community organizations serving PWUD, such as OAT programs and shelters, they were more hesitant to rely on other government-supported healthcare services, including public hospitals. In addition, they pointed to the limited availability of resources for medical professionals, which they felt hindered the provision of accurate and timely interventions, attributing this challenge primarily to perceived inefficiencies within the Greek government, which is largely responsible for healthcare provision. In contrast, participants relayed that they trust the staff at community organizations with extension of that trust to healthcare provided through these settings. Biancarelli et al*.* [28] also found that PWUD formed stronger connections with the staff of community organizations compared to large healthcare institutions. These findings suggest that community organizations can enhance trust in, and promotion of interventions designed to address PWUDs’ healthcare issues.

In addition to traditional methods of accessing healthcare in Greece, such as scheduling appointments and utilizing emergency medical services, our participants also used the internet to identify healthcare providers and book in-person appointments. Healthcare delivery through the internet is an innovative approach to improve healthcare access. Telehealth is an umbrella term that includes telemedicine-based virtual visits, chat-based patient-provider interactions, and patient monitoring at a distance [29]. Recent evidence indicates that telehealth could be one approach to expand healthcare access for PWUD, especially when integrated into OAT programs for HCV treatment [30–36]. Telehealth’s role, however, as a tool in expanding healthcare access among PWUD for a broad range of conditions and settings, not limited to OAT programs, requires further investigation. To address this research gap, investigators must initially understand the process of how PWUD access existing healthcare institutions. Such an understanding could provide a framework within which telehealth may be applied among the PWUD population and potentially integrated into existing healthcare institutions that primarily support PWUD [37].

Our relatively large sample size for FGD studies enhances the rigor of the results [38, 39]. The large sample size also facilitated attainment of theoretical saturation [19]. Achieving saturation enhanced the analytic depth and precision of our study [19]. Additionally, we established an analysis team that represented diverse specialties, where each analyst engaged in independent coding, that enhanced the findings’ credibility. Furthermore, through team discussion and consensus, we potentially mitigated researcher-associated bias [40, 41]. However, researcher-associated bias cannot be entirely ruled out. Another potential limitation, inherently related to the FGD design, is bias related to the participants’ reported experiences and the potential for group think [42]. We attempted to mitigate any potential bias by encouraging each participant to actively contribute to the discussion. Moreover, our participants were predominantly males of Greek origin, which could impact the transferability of the findings to other populations [40]. For example, our findings may not fully capture the experiences and barriers related to healthcare access among specific PWUD subgroups, such as women, migrants, and ethnic minorities. For instance, SUD among women has been associated with heavier stigma and childcare responsibilities that can further complicate their ability to seek healthcare [43]. Among migrant PWUD, cultural competency barriers, including language and legal concerns, have been described [44, 45]. The lack of inclusion of substantial numbers of female and migrants may have impacted our ability to identify these specific issues. Lastly, and despite our inclusion efforts, we may not have captured every participant’s perspective within the FGDs [46].

## Conclusions

PWUD in Athens, Greece, report seeking healthcare most frequently when experiencing critical, life-threatening situations. Their healthcare journey is challenged by numerous obstacles including stigma in the healthcare setting, perceived poor healthcare quality, systematic delays related to inadequate response of emergency medical services, and mistrust in healthcare providers and institutions. To address these challenges and improve healthcare access for PWUD, we suggest the following recommendations:

- **Minimize stigma and promote empathy in healthcare encounters**: Healthcare professionals should treat PWUD equitably, recognizing SUD as a medical condition. Prioritizing empathy and respect in healthcare encounters can mitigate stigma and discrimination, foster a supportive environment, and enhance the quality of care.
- **Enhance healthcare staff education on SUD**: Provide adequate education for healthcare staff on SUD, including comprehensive training on withdrawal management. The education should also emphasize awareness of coexisting medical and social factors impacting PWUD, and the role of healthcare professionals in addressing these challenges to provide comprehensive medical care.
- **Improve emergency medical services responsiveness**: Enhance the responsiveness of emergency medical services to calls involving PWUD, ensuring accurate and timely care in critical situations. Moreover, emergency services often serve as the primary point of contact with the healthcare system for many PWUD, offering valuable opportunities to engage them in necessary healthcare and social support.
- **Build trust through community engagement**: Collaborate with community organizations that serve PWUD, building on the trust already established in these settings, to bridge the gaps between PWUD and the healthcare system. Enhance healthcare delivery directly at these community sites and foster partnerships between these organizations, healthcare providers, and institutions. By building upon the foundation of trust, healthcare engagement and outcomes can be significantly improved.
- **Explore telehealth’s role in improving healthcare access for PWUD**: Telehealth holds promise in expanding healthcare access for PWUD, with potential applications at community organizations, at OAT programs, and in other settings. Engaging PWUD in the design and delivery of telehealth-based interventions may ensure their relevance and enhance effectiveness.

These participant-derived recommendations offer key strategies to improve healthcare access for PWUD and should be considered by healthcare providers, researchers, and policymakers when designing interventions aimed at addressing the unique healthcare access challenges faced by this population.

## Supplementary Information

Additional file 1.

Additional file 2.

## Data Availability

The FGD transcripts and analyses are available from the corresponding author upon reasonable request.

## Abbreviations

PWUD: People who use drugs
HCV: Hepatitis C virus
FGDs: Focus group discussions
SUD: Substance use disorder
OAT: Opioid agonist treatment
