# Supplementary material for: Breaking the vicious cycle of delayed healthcare seeking for people who use drugs
Source: Harm Reduct J. 2025 Mar 5;22:27. doi: 10.1186/s12954-025-01166-3 (PMC11881266; doi:10.1186/s12954-025-01166-3)
Supplement: Supplementary file 1 — Additional file 1. [file 12954_2025_1166_MOESM1_ESM.docx]

**Additional file 1.** Interview guide for the focus group discussions

- Please describe your personal experiences with the current health care system.
- How satisfied (or not) are you with the healthcare you receive?
- Please tell me about some of those experiences.
- Please describe how do you find a doctor when you have a medical problem.
- Tell me a story about how you received medical care when you were sick.
- Please tell me about the process of accessing healthcare.
- Where do you (or not) access healthcare (e.g., doctor’s office, hospital, health care center, emergency department, substance use treatment program)?
- How do you learn about health information?
- Tell me a story about how you gain health information.
- What are your expectations for high-quality healthcare?
- What are the things that you value in a healthcare provider?
- What are things that you don’t like about healthcare providers?
- Please tell me your feelings about trusting the current healthcare system in terms of respecting your confidentiality, privacy, and security?
- Please describe the challenges and barriers that interfered with your ability to obtain medical care.
- How do you overcome these barriers?
- Do you have anything to add that we haven’t talked about?
