# Supplementary material for: Breaking the vicious cycle of delayed healthcare seeking for people who use drugs
Source: Harm Reduct J. 2025 Mar 5;22:27. doi: 10.1186/s12954-025-01166-3 (PMC11881266; doi:10.1186/s12954-025-01166-3)
Supplement: Supplementary file 2 — Additional file 2. [file 12954_2025_1166_MOESM2_ESM.docx]

**Additional file 2.** Consolidated criteria for reporting qualitative research (COREQ) Checklist.

The table below illustrates how and where (when applicable) our study addresses each item in the COREQ checklist.

| **Characteristic** | **Guide questions/description** | **Manuscript page number** | **Description or relevant text from manuscript.** |
| --- | --- | --- | --- |
| **Domain 1: Research team and reflexivity** | | | |
| *Personal Characteristics* | | | |
| 1. Interviewer/facilitator: | Which author/s conducted the interview or focus group? | Manuscript, page 8 | “The FGDs were conducted by the research study manager (Z.P.) along with the co-principal investigator (A.H.), who had received training in qualitative interviewing and FGD coordination from an expert in qualitative research (S.S.D.)”. |
| 2. Credentials | What were the researcher’s credentials? E.g. PhD, MD |  | Z.P.: MD  A.H.: MD, PhD |
| 3. Occupation | What was their occupation at the time of the study? |  | Z.P. is the Research Support Specialist/Research Project Manager.  A.H. is Professor Emeritus of Epidemiology and Preventive Medicine at the National and Kapodistrian University of Athens Medical School.  He has served on many Executive Committees including the presidency of the Hellenic Centers for Disease Control. He is Co-Chair and founder of the Hepatitis B and C Public Policy Association. |
| 4. Gender | Was the researcher male or female? |  | Z.P. is female and A.H. is male. |
| 5. Experience and training | What experience or training did the researchers have? | Manuscript, pages 8-10 | Zoi Papalamprakopoulou^1,2^, Arpan Dharia^1^, Suzanne S. Dickerson^3^, Angelos Hatzakis^2,4^, Andrew Talal^1^  ^1^Division of Gastroenterology, Hepatology, and Nutrition, Jacobs School of Medicine and Biomedical Sciences, University at Buffalo, Buffalo, NY, United States  ^2^Hellenic Scientific Society for the Study of AIDS, Sexually Transmitted and Emerging Diseases, Athens, Greece,  ^3^Faculty Development and PhD Program, School of Nursing, University at Buffalo, Buffalo, NY, United States  ^4^Department of Hygiene, Epidemiology and Medical Statistics, National and Kapodistrian University of Athens, Athens, Greece  S.S.D. is an expert in qualitative research and has been a member of the Advanced Hermeneutical Institute for the past three decades. She utilizes in PhD training coursework, the definitive book on the topic entitled “Constructing Grounded Theory” (2014, Sage Publication Inc., ISBN 978-1-4462-9349-2). S.D. provided training to Z.P. and A.H. through lectures, access to academic materials, and practical mock interviews to facilitate appropriate focus group conduction.  “The FGDs were conducted by the research study manager (Z.P.) along with the co-principal investigator (A.H.), who had received training in qualitative interviewing and FGD coordination from an expert in qualitative research (S.S.D.)”.  “The initial analysis team consisted of the research study manager (Z.P.), co-investigator (A.D.), and the principal investigator (A.H.T.), who conducted the initial thematic coding and analysis of the FGD transcripts and accompanying field notes. Subsequently, an expert in qualitative methodology (S.S.D.), along with the co-principal investigator (A.H) joined the final analysis team to offer their opinions on the appropriateness of the analysis. The co-principal investigator possesses extensive cultural expertise from significant research experience working with PWUD”. |
| *Relationship with participants* | | | |
| 6. Relationship established | Was a relationship established prior to study commencement? |  | No. |
| 7. Participant knowledge of the interviewer | What did the participants know about the researcher? e.g. personal goals, reasons for doing the research- |  | Participants were not aware beforehand of the researchers. However, all participants in the FGD were aware of the research's intent, either through communication with the facilitators or direct contact with the research study manager. Ultimately, participants were informed of the purpose of the investigation during the informed consent process. |
| 8. Interviewer characteristics | What characteristics were reported about the interviewer/facilitator? e.g. Bias, assumptions, reasons and interests in the research topic |  | Z.P. explained that she is an MD and has been working as the Research Support Specialist/Research Study Manager on the study for several months. She elaborated that her initial involvement with the study team began in 2022 after expressing her interest in collaborating with A.T. and his team to explore optimal healthcare strategies for people who use drugs.  A.H. explained that he is a professor emeritus who has been collaborating with the study team for several months. He explained that throughout his career, he has been dedicated to studying and planning interventions for the treatment of infectious diseases among people who use drugs. |
| **Domain 2: Study design** | | | |
| *Theoretical framework* | | | |
| 9. Methodological orientation and Theory | What methodological orientation was stated to underpin the study? e.g. grounded theory,  discourse analysis, ethnography, phenomenology, content analysis | Manuscript, page 9 | “We employed modified grounded theory as the most suitable approach to analyze the FGDs and identify the specific processes that PWUD use to access healthcare. Employing a modified grounded theory approach, we integrated existing theoretical knowledge about the challenges experienced by PWUD in accessing healthcare, as identified in prior research, while maintaining flexibility to allow new themes to emerge through data analysis [18-21]”. |
| *Participant selection* | | | |
| 10. Sampling | How were participants selected? e.g. purposive, convenience, consecutive, snowball | Manuscript page 7 | “We employed purposive sampling to recruit participants [12, 13]”. “Inclusion criteria for participation included age ≥18 years, history of injection drug use, current internet access, Greek verbal fluency, and the ability to provide informed consent”. |
| 11. Method of approach | How were participants approached? e.g. face-to-face, telephone, mail, email | Manuscript, page 7 | “We contacted influential colleagues from community organizations that serve PWUD, including the Hellenic Liver Patient Association, “Prometheus”, “My Athens” (a shelter for PWUD experiencing homelessness), “Positive Voice” (an association of people living with HIV), and the “Network of Peer Users of Psychoactive Substances” to serve as facilitators for participant recruitment. The facilitators were employed by the aforementioned community organizations and possessed bachelor’s degrees in psychology or social work. The facilitators were educated on the details of the study by the research staff. We identified prospective participants through their responses to in-person announcements or through social media posts disseminated by the community organizations. The research study manager (Z.P.) contacted potential participants, either directly or through a warm hand-off process mediated by a facilitator”. |
| 12. Sample size | How many participants were in the study? | Manuscript, page 8 | **“**Between May and September 2023, we conducted 9 FGDs, each comprised of 4 to 9 participants (N=57).” |
| 13. Non-participation | How many people refused to participate or dropped out? Reasons? | Manuscript, page 7,8 | “We identified prospective participants through their responses to in-person announcements or through social media posts disseminated by the community organizations.” Hence, it was not possible to calculate how many participants may have refused to participate in the study, as all individuals who contacted the research team through social media announcements were eligible and willing to participate. “There were no participants who prematurely discontinued the FGD”. |
| *Setting* | | | |
| 14. Setting of data collection | Where was the data collected? e.g. home, clinic, workplace | Manuscript, page 8 | “Each FGD lasted up to 90 minutes and took place in a private room in the premises of “Prometheus”. |
| 15. Presence of non-participants | Was anyone else present besides the participants and researchers? | N/A | Only the participants and the researchers were present at the time of the FGD. |
| 16. Description of sample | What are the important characteristics of the sample? e.g. demographic data, date. | Manuscript, page 11 | “Participants had a mean age with a standard deviation of 47.9 (8.9) years and 89.5% (51/57) were male. The majority (91.2%, 52/57) were of Greek origin and 61.4% (35/57) had attended at least 10 years of school.” |
| *Data collection* | | | |
| 17. Interview guide | Were questions, prompts, guides provided by the authors? Was it pilot tested? | Manuscript, page 8  Additional file 1 | “We developed a semi-structured interview guide containing open-ended, non-leading questions to facilitate discussions about healthcare access. Example questions included: “Please describe how do you find a doctor when you have a medical problem?”, “Please tell me about the process of accessing healthcare?”, and “Please describe the challenges and barriers that interfered with your ability to obtain medical care”. An additional file contains the complete interview guide [see Additional file 1]. If participants did not mention these aspects during the open-ended inquiry, we subsequently employed probes to further elaborate on their approaches to accessing healthcare.”  “Prior to deployment of the interview guide, we asked the study facilitators for their critical appraisal of the cultural and literary relevance of the interview guide for our study population. All facilitators agreed on the guide’s comprehensibility and appropriateness. We also pilot tested the interview guide in one pilot FGD with four study-eligible participants, following the planned study process for recruitment, enrollment, and informed consent. After the pilot FGD, we made minor literary modifications to the interview guide to ensure further clarity and comprehensibility by the study population”.  An additional file contains the complete interview guide [see Additional file 1]. |
| 18. Repeat interviews | Were repeat interviews carried out? If yes, how many? | N/A | No repeat interviews were performed. |
| 19. Audio/visual recording | Did the research use audio or visual recording to collect the data? | Manuscript, page 9 | “The FGDs were audio-recorded, transcribed verbatim, and translated into English by a professional agency. Field notes were also made during the FGD” |
| 20. Field notes | Were field notes made during and/or after the interview or focus group? | Manuscript, page 9 | “Field notes were also made during the FGD.” The field notes were incorporated into the analytic process. |
| 21. Duration | What was the duration of the interviews or focus group? | Manuscript, page 8 | “Each FGD lasted up to 90 minutes and took place in a private room in the premises of “Prometheus”. |
| 22. Data saturation | Was data saturation discussed? | Manuscript, page 10 | “When no new insights emerged from the analysis of the FGD transcripts, we determined that we had achieved saturation and concluded further data collection”. |
| 23. Transcripts returned | Were transcripts returned to participants for comment and/or correction? | Manuscript, page 9 | The transcripts were not returned to the participants for comment and/ or correction. “The FGD transcripts were verified for accuracy by the research project manager and interviewer (Z.P.) through comparison with the recordings” |
| **Domain 3: analysis and findings** | | | |
| *Data analysis* | | | |
| 24. Number of data coders | How many data coders coded the data? | Manuscript, page 10 | “The initial analysis team consisted of the research study manager (Z.P.), co-investigator (A.D.), and the principal investigator (A.H.T.), who conducted the initial thematic coding and analysis of the FGD transcripts and accompanying field notes. Subsequently, an expert in qualitative methodology (S.S.D.), along with the co-principal investigator (A.H) joined the final analysis team to offer their opinions on the appropriateness of the analysis. The co-principal investigator possesses extensive cultural expertise from significant research experience working with PWUD”. |
| 25. Description of the coding tree | Did authors provide a description of the coding tree? | Manuscript, page 10 | “The iterative analysis process followed a deductive approach, involving initial independent coding by each analyst, which included preliminary themes and initial quotes. The initial analysis team consisted of the research study manager (Z.P.), co-investigator (A.D.), and the principal investigator (A.H.T.), who conducted the initial thematic coding and analysis of the FGD transcripts and accompanying field notes. Subsequently, an expert in qualitative methodology (S.S.D.), along with the co-principal investigator (A.H) joined the final analysis team to offer their opinions on the appropriateness of the analysis. The co-principal investigator possesses extensive cultural expertise from significant research experience working with PWUD. These findings were subsequently shared among the members of the qualitative data analysis team during weekly meetings. During these meetings, the analysts reviewed the FGD transcripts and field notes, discussed their findings, and compared them with previous transcripts. Any disagreements between the analysts were discussed until consensus was reached; if no consensus was reached, the original transcript text was revisited. The full five-member analysis team conducted the final coalescence of themes. When no new insights emerged from the analysis of the FGD transcripts, we determined that we had achieved saturation and concluded further data collection”. |
| 26. Derivation of themes | Were themes identified in advance or derived from the data? | Manuscript, page 9, 10 | “We employed modified grounded theory as the most suitable approach to analyze the FGDs and identify the specific processes that PWUD use to access healthcare. Employing a modified grounded theory approach, we integrated existing theoretical knowledge about the challenges experienced by PWUD in accessing healthcare, as identified in prior research, while maintaining flexibility to allow new themes to emerge through data analysis [18-21]”.  “These findings were subsequently shared among the members of the qualitative data analysis team during weekly meetings. During these meetings, the analysts reviewed the FGD transcripts and field notes, discussed their findings, and compared them with previous transcripts. Any disagreements between the analysts were discussed until consensus was reached; if no consensus was reached, the original transcript text was revisited”. |
| 27. Software | What software, if applicable, was used to manage the data? | N/A |  |
| 28. Participant checking | Did participants provide feedback on the findings? | N/A | The participants of the FGDs did not provide feedback on the findings. Nevertheless, we have shared initial findings of the analysis with the study facilitators. Both the facilitators and the analysis team were individuals who had extensive experience working with the opioid use disorder patient population. All members of the analysis team agree with the findings as outlined in the submitted manuscript. |
| *Reporting* | | | |
| 29. Quotations presented | Were participant quotations presented to illustrate the themes / findings? Was each quotation identified? e.g. participant number | Manuscript, pages 11-19, Figure 1 | Yes, representative participant quotations are presented to illustrate the findings and are labeled with a unique participant number to protect participant anonymity. |
| 30. Data and findings consistent | Was there consistency between the data presented and the findings? | Manuscript, pages 11-19,  Figure 1 | Yes, the themes serve as evidence of the study findings, and the overarching core category explains the underlying processes that support those findings. |
| 31. Clarity of major themes | Were major themes clearly presented in the findings? | Manuscript, pages 11-18,  Figure 1 | Yes, the major themes are clearly presented in the results section, accompanied by example participant quotations.  “We identified three key themes from the FGD transcript analysis: (1) seeking care after an individual’s rapid health decline, (2) facing barriers in accessing healthcare, and (3) building trust to improve access to healthcare for PWUD. Figure 1 illustrates the key themes that emerged from the FGDs (Figure 1)”. |
| 32. Clarity of minor themes | Is there a description of diverse cases or discussion of minor themes? | Manuscript, pages 11-18, | Yes, we have described diverse cases and minor themes throughout the results section. |

Tong A, Sainsbury P, Craig J. Consolidated criteria for reporting qualitative research (COREQ): a 32-item checklist for interviews and focus groups. International Journal for Quality in Health Care. 2007;19(6):349-57
